# Esketamine-sufentanil PCA reduces postoperative depression state in elderly colorectal cancer patients: a randomized controlled trial

**DOI:** 10.1038/s41598-026-49287-4

**Published:** 2026-04-21

**Authors:** Rong Ding, Daojie Wang, Guoxin Wang, Yamei Lin, Dongchen Wu, Min Zhang, Yufei Ren, Wenyue Kang, Guanwen Lin

**Affiliations:** Department of Anesthesiology, Hainan General Hospital, Hainan Affiliated Hospital of Hainan Medical University, Haikou, Hainan China

**Keywords:** Esketamine, Postoperative depression state, Elderly, Colorectal cancer, Patient-controlled analgesia, Cancer, Drug discovery, Health care, Medical research, Oncology

## Abstract

Elderly colorectal cancer (CRC) patients face high risks of postoperative depression state, inadequately addressed by opioid-based analgesia. Esketamine, an NMDA receptor antagonist with rapid antidepressant effects, offers potential benefits but lacks evidence in this population. This double-blind RCT enrolled 99 elderly (≥ 65 years) CRC resection patients, randomized to three postoperative PCA groups: C: Sufentanil (2 µg/kg) + saline placebo, ES1: Sufentanil + esketamine 1 mg/kg, ES2: Sufentanil + esketamine 2 mg/kg. Primary outcomes were anxiety/depression (HAMA/HAMD scores) at 24 h postoperatively. Secondary outcomes included VAS pain scores, patient satisfaction, and adverse events. Depression/Anxiety: Both esketamine groups showed significantly lower HAMD/HAMA scores vs. control at 24 h and 72 h (e.g., 24 h HAMD: ES1 6.16 ± 2.16, ES2 7.10 ± 2.55 vs. C 9.87 ± 3.67; *p* < 0.001), with no dose-dependent difference (*p* > 0.05 ES1 vs. ES2). Pain Control: No intergroup differences in resting/activity VAS scores or rescue analgesia demands (*p* > 0.05). Satisfaction: Higher satisfaction rates in ES1 (77.4%) and ES2 (90.0%) vs. C (50.0%) (*p* = 0.002). Safety: Comparable adverse events across groups (*p* > 0.05). Low-dose esketamine (1 mg/kg) adjunct to sufentanil PCA significantly reduces postoperative depression and anxiety state in elderly CRC patients without enhancing analgesia or increasing adverse events. Its profound psychological benefits and high patient satisfaction support its integration into enhanced recovery protocols for this vulnerable cohort. Registry: Chinese Clinical Trial Registry(chictr.org.cn), TRN: ChiCTR2300070763, Registration date: 23 April 2023.

## Introduction

Colorectal cancer (CRC) ranks as the third most commonly diagnosed malignancy and the second leading cause of cancer-related deaths globally, with an estimated 1.93 million new cases and 916,000 deaths annually^1^. Notably, over 50% of CRC patients are aged ≥ 70 years, and this proportion continues to rise with accelerating population aging^2–4^. Surgical resection remains the cornerstone of curative treatment for localized CRC. However, elderly patients face disproportionately high risks of postoperative complications, including infections, cardiovascular events, and prolonged recovery, which are strongly associated with reduced long-term survival^5^. Beyond physical morbidity, psychological distress—particularly postoperative depression state—has emerged as a critical yet underaddressed challenge.

Conventional postoperative pain management relies heavily on opioid-based patient-controlled intravenous analgesia (PCIA). While effective for pain relief, opioids carry significant risks in elderly populations, including respiratory depression, ileus, and cognitive impairment^6^. Moreover, opioids lack intrinsic antidepressant properties and may exacerbate depressive symptoms through mechanisms such as dysregulation of the hypothalamic-pituitary-adrenal axis^7^. Non-opioid adjuncts, such as regional anesthesia or nonsteroidal anti-inflammatory drugs (NSAIDs), are limited by variable efficacy or contraindications in frail patients^8^. Thus, there is an urgent need for analgesic strategies that simultaneously address pain and psychological morbidity in elderly CRC patients.

Esketamine, the S(+)-enantiomer of ketamine, is a non-competitive N-methyl-D-aspartate (NMDA) receptor antagonist with rapid-acting antidepressant effects. Unlike traditional antidepressants requiring weeks to take effect, esketamine modulates glutamatergic neurotransmission within hours by blocking NMDA receptors on GABAergic interneurons, thereby disinhibiting prefrontal cortical pyramidal neurons and enhancing synaptic plasticity via brain-derived neurotrophic factor (BDNF) signaling^9,10^. Clinically, subanesthetic doses of esketamine have demonstrated efficacy in reducing depressive symptoms in patients with treatment-resistant depression and cancer-related psychological distress. Additionally, its opioid-sparing properties and minimal respiratory depression make it particularly appealing for geriatric perioperative care.

Despite these advantages, evidence on esketamine’s role in postoperative depression state among elderly CRC patients remains scarce. Existing studies predominantly focus on younger cohorts or non-gastrointestinal surgeries, with limited exploration of dose-response relationships or long-term outcomes^11^. Furthermore, the interplay between esketamine’s antidepressant effects and its analgesic efficacy in this population is poorly understood. This knowledge gap hinders the optimization of perioperative protocols tailored to the unique needs of elderly CRC patients.

The present study aims to evaluate the impact of esketamine, administered via PCIA in combination with sufentanil, on postoperative depression and anxiety state in elderly patients undergoing CRC resection. By comparing two esketamine doses (1 mg/kg and 2 mg/kg) against a sufentanil-only control, we seek to elucidate its dose-dependent effects on mood regulation, pain control, and patient satisfaction. Our findings may inform the development of integrated perioperative strategies that enhance both physical and psychological recovery in this vulnerable population.

## Materials and methods

### Study design and ethics

This prospective, double-blind, randomized controlled trial (RCT) was approved by the Ethics Committee of Hainan General Hospital (Approval No. [2022]703) and registered at Chinese Clinical Trial Registry (ID: ChiCTR2300070763). Written informed consent was obtained from all participants.

### Participants

#### Inclusion criteria

Elderly patients (≥ 65 years) scheduled for elective radical colorectal cancer surgery between May 1, 2023 and January 1, 2024 with:

1. ASA physical status I–II.
2. Capacity to comprehend study procedures and provide voluntary consent.

#### Exclusion criteria

1. Refusal to participate.
2. Contraindications to general anesthesia or prior anesthesia-related complications.
3. Allergy/hypersensitivity to esketamine, propofol, opioids, or neuromuscular blocking agents.
4. Uncontrolled hypertension (SBP > 180 mmHg), hypotension (SBP < 90 mmHg), or diabetes (HbA1c > 8%).
5. History of alcohol/drug dependence.
6. Chronic use of analgesics/sedatives (> 3 months).
7. Pre-existing psychiatric disorders (e.g., depression, schizophrenia).
8. Participation in other trials within 2 months.
9. Investigator-judged clinical unsuitability.

### Sample size calculation

The sample size was determined a priori using G*Power software (Version 3.1.9.2; Heinrich-Heine-Universität Düsseldorf, Germany). Based on the results of the pilot data (with *n* = 10 participants per group), an effect size of Cohen’s f = 0.333 was adopted as the primary input parameter. The calculation was configured for a three-independent-group comparative design, with a significance level (α) of 0.05 and a statistical power (1–β) of 0.80.

The analysis indicated that a total of 90 participants (30 per group) would be required to detect a statistically significant difference among the groups. Considering an anticipated attrition rate of approximately 10% during the follow-up period, a total of 99 participants (33 per group) will be recruited.

### Randomization, blinding, and intervention

#### Randomization

99 participants were simply randomized by computer to the following three groups (Table 1):

**Table 1:** Intervention groups.

Group	Intervention	*n*
C	Placebo (normal saline)	33
ES1	Esketamine 1 mg/kg* + Sufentanil	33
ES2	Esketamine 2 mg/kg* + Sufentanil	33

*Diluted in 150 ml saline; Sufentanil 2 µg/kg for all groups.

#### Blinding protocol

- Drug preparation: Solutions prepared in identical opaque bags by independent pharmacist.
- Anesthesia management: Performed by blinded anesthesiologist.
- Outcome assessment: HAMA/HAMD administered preoperatively (24 h) and postoperatively by blinded researcher.

### Anesthesia protocol

#### Standardized procedures

- Monitoring: ECG, SpO_2_, BIS, invasive BP.
- Preoxygenation: 100% O_2_ (6 L/min × 5 min).
- Induction: Etomidate 0.2 mg/kg + Sufentanil 0.5 µg/kg + Cisatracurium 0.2 mg/kg.
- Maintenance: Propofol 4–5 mg/kg/h + Remifentanil 0.1–0.2 µg/kg/min + Cisatracurium PRN.
- Vital targets: HR > 50 bpm, SpO_2_ 95–100%, ETCO_2_ 35–45 mmHg, BIS 40–60.

#### Postoperative analgesia (PCA)

- Continuous infusion: 0.5 ml/h.
- Bolus: 2 ml (lockout 15 min) (Table 2).

**Table 2:** Outcome measures.

Category	Parameter	Timepoint
Primary	Anxiety/depression (HAMA/HAMD score ≥ 8)	24 h postoperatively
Secondary	VAS pain score	2 h, 6 h, 24 h,72hpostop
	Supplemental analgesia demands	0–24 h postop
	Patient satisfaction (0–10 scale)	72 h postop
	Hospital stay (days)	Discharge
Safety	PONV, dizziness, nightmares	0–72 h postop
	Nystagmus, pruritus, hallucinations	-

### Statistical analysis

#### Software: SPSS 26.0 (IBM Corp., Armonk, NY)

##### Methods

- Continuous data: Mean ± SD.
- Between-group (single timepoint): One-way ANOVA + Bonferroni.
- Repeated measures: Two-way RM-ANOVA + within-group contrast analysis.
- Categorical data: n (%).
- Chi-square or Fisher’s exact test.
- Sample size: A total of ninety-nine patients will be randomly allocated to three groups (C, ES1, and ES2) with 33 patients in each group.
- Correlation analysis: Kendall’s tau-b correlation was used to examine the relationship between 24‑h postoperative VAS (rest and activity) and HAMD scores in the total population and within each group.

Significance *p* < 0.05 (two-tailed).

**Figure:**
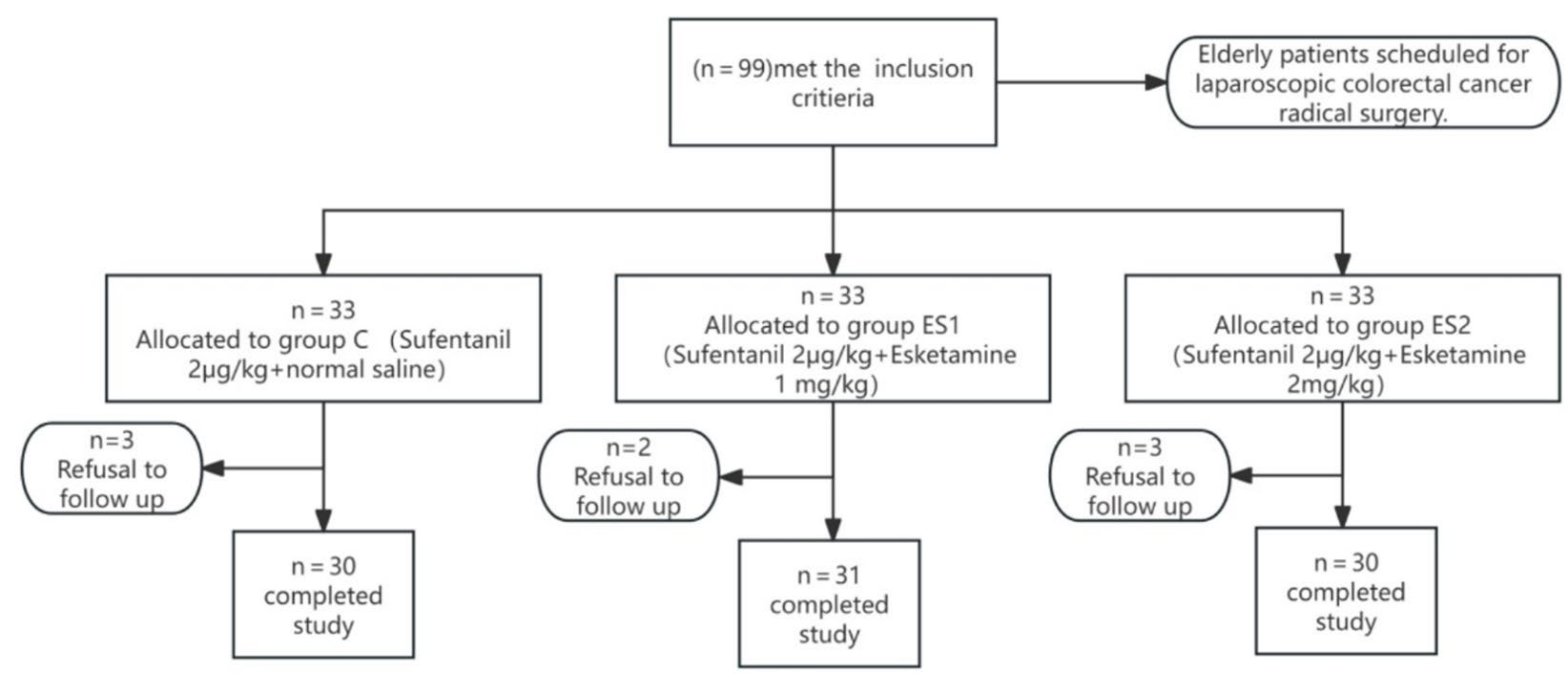
CONSORT flowchart.

## Results

### Baseline characteristics

A total of 99 patients were enrolled and randomized. Of these, 8 were excluded from the final analysis: 5 patients withdrew consent or had family members refuse assessment during the preoperative evaluation on the day before surgery, and 3 patients were excluded due to intraoperative conversion to alternative surgical procedures. Importantly, none of these patients received the study intervention.By excluding only patients who did not receive the intervention, we minimized the risk of introducing systematic bias into the analysis.

No significant differences were observed among the three groups in gender, age, BMI, motion sickness history, or education level (*p* > 0.05, Table 3).

**Table 3:** Demographic and clinical characteristics.

Characteristic	Group C (*n* = 30)	Group ES1 (*n* = 31)	Group ES2 (*n* = 30)	F/χ^2^	*p*-value
Male/Female, n	15/15	20/11	16/14	1.817	0.403
Age, years	70.6 ± 4.5	71.8 ± 4.3	71.2 ± 4.9	0.531	0.590
Height, cm	160.8 ± 8.1	161.6 ± 7.7	158.8 ± 7.5	1.033	0.360
Weight, kg	56.6 ± 9.8	56.9 ± 10.8	52.6 ± 9.1	1.740	0.182
BMI, kg/m^2^	21.8 ± 3.0	21.7 ± 3.0	20.8 ± 2.9	0.013	0.367
Motion Sickness, n (%)	6 (20.0)	6 (19.4)	7 (23.3)	2.371	0.668
Education Level, n (%)				0.189	0.910
Illiteracy (< 1 year)	4 (13.3)	6 (19.4)	4 (13.3)		
Primary (1–6 years)	7 (23.3)	4 (12.9)	8 (26.7)		
Middle (6–9 years)	8 (26.7)	7 (22.6)	5 (16.7)		
High (9–12 years)	9 (30.0)	11 (35.5)	12 (40.0)		
University (> 12 years)	2 (6.7)	3 (9.7)	1 (3.3)		

### Depression and anxiety scores

#### HAMD scores (depression)

**Fig. 1:**
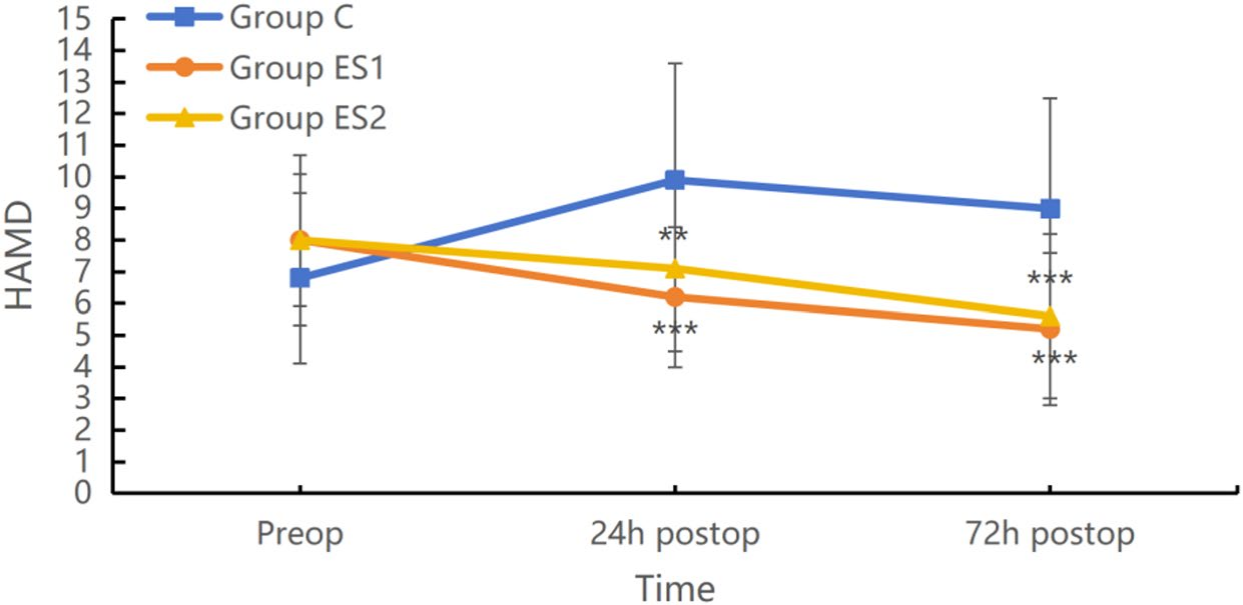
Longitudinal changes of HAMD-17 scores in the three groups. ***p < 0.001, **p < 0.01 vs. Group C at the same timepoint.

The longitudinal changes in HAMD-17 scores across the three groups are illustrated in Fig. 1. At baseline (Preop), no significant differences in HAMD-17 scores were observed among the three groups (*p* = 0.094). In the control group (Group C), HAMD-17 scores increased from 6.8 ± 2.7 at baseline to 9.9 ± 3.7 at 24 h postoperatively, and slightly decreased to 9.0 ± 3.5 at 72 h postoperatively. In contrast, both esketamine groups (ES1 and ES2) showed downward trend in HAMD-17 scores after surgery.

#### HAMA scores (anxiety)

**Fig. 2:**
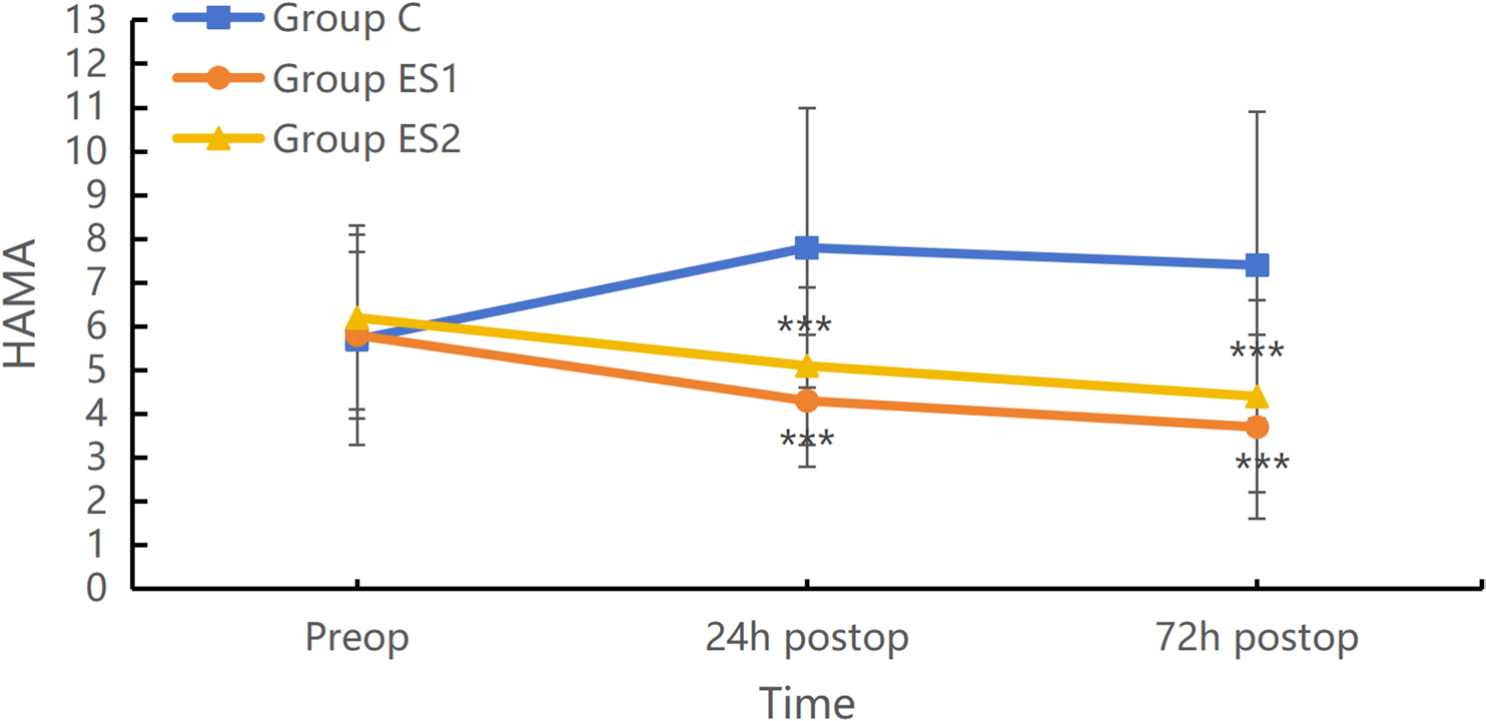
Longitudinal changes of HAMA scores in the three groups. ***p < 0.001 vs. Group C at the same timepoint.

The longitudinal changes in Hamilton Anxiety Rating Scale (HAMA) scores across the three groups are illustrated in Fig. 2. At baseline (Preop), there were no significant differences in HAMA scores among Group C, Group ES1, and Group ES2 (5.7 ± 2.4 vs. 5.8 ± 1.9 vs. 6.2 ± 2.1, respectively; *p* = 0.581).

In the control group (Group C), HAMA scores increased from 5.7 ± 2.4 at baseline to 7.8 ± 3.2 at 24 h postoperatively, and remained elevated at 7.4 ± 3.5 at 72 h postoperatively. In contrast, both esketamine-treated groups showed a sustained reduction in HAMA scores after surgery.For comprehensive details of both HAMD and HAMA scores, including all timepoint values, statistical tests, and pairwise comparisons, please refer to Table 4.

**Table 4:** Longitudinal changes in depression and anxiety scores.

Scale	Timepoint	Group C	Group ES1	Group ES2	F or χ^2^	*p*-value	Pairwise *p* (vs. C)
HAMD	Preop	6.8 ± 2.7	8.0 ± 2.1	8.0 ± 2.7	2.424	0.094	-
	24 h postop	9.9 ± 3.7	6.2 ± 2.2	7.1 ± 2.6	56.099	< 0.001	< 0.001 (ES1), 0.004 (ES2)
	72 h postop	9.0 ± 3.5	5.2 ± 2.4	5.6 ± 2.6	16.189	< 0.001	< 0.001 (both)
HAMA	Preop	5.7 ± 2.4	5.8 ± 1.9	6.2 ± 2.1	0.546	0.581	-
	24 h postop	7.8 ± 3.2	4.3 ± 1.5	5.1 ± 1.8	54.159	< 0.001	< 0.001 (both)
	72 h postop	7.4 ± 3.5	3.7 ± 2.1	4.4 ± 2.2	16.620	< 0.001	< 0.001 (both)

### Postoperative pain scores

#### VAS at rest

Significant time effect (*F* = 109.98, *p* < 0.001) but no group or interaction effects (Table 5).

**Table 5:** Resting VAS scores.

Group	2 h postop	6 h postop	24 h postop	72 h postop	*p* (Time)
C	2.10 ± 1.01^a^	1.62 ± 1.21^a^	0.62 ± 0.82^b^	0.34 ± 0.55^b^	< 0.001
ES1	2.13 ± 1.15^a^	1.65 ± 1.14^a^	0.71 ± 0.82^b^	0.32 ± 0.54^b^	< 0.001
ES2	2.10 ± 0.99^a^	2.10 ± 0.99^a^	0.97 ± 1.00^b^	0.37 ± 0.67^b^	< 0.001

^ab^Different superscripts indicate significant within-group differences (*p* < 0.05).

Group Effect: *F* = 0.872, *p* = 0.422; Interaction: *F* = 0.941, *p* = 0.454.

#### VAS during activity

Significant time effect (*F* = 51.903, *p* < 0.001) but no group or interaction effects (Table 6).

**Table 6:** Activity VAS scores.

Group	2 h postop	6 h postop	24 h postop	72 h postop	*p* (Time)
C	3.48 ± 0.95^bc^	4.00 ± 1.28^b^	2.93 ± 1.00^c^	2.31 ± 1.20^a^	< 0.001
ES1	3.35 ± 1.74^bc^	3.81 ± 1.49^b^	2.87 ± 1.34^c^	1.97 ± 0.80^a^	< 0.001
ES2	3.73 ± 1.44^bc^	4.07 ± 1.36^b^	3.17 ± 1.39^c^	2.07 ± 0.83^a^	< 0.001

^abc^Different superscripts indicate significant within-group differences (*p* < 0.05).

Group Effect: *F* = 0.681, *p* = 0.509; Interaction: *F* = 0.365, *p* = 0.846.

#### PCA bolus demands

No significant difference: C: 4.69 ± 4.38, ES1: 4.16 ± 2.60, ES2: 5.77 ± 6.59 (*F* = 0.884, *p* = 0.417).

### Safety and patient-reported outcomes

#### Adverse events

See Table 7.

**Table 7:** Adverse events and hospital stay.

Group	24 h AE, *n* (%)	48 h AE, *n* (%)	Postop LOS (days)
C	8 (26.7)	6 (20.0)	8.67 ± 3.61
ES1	7 (22.6)	1 (3.2)	8.80 ± 2.34
ES2	5 (16.7)	3 (10.0)	9.85 ± 4.48
*p*	0.643	0.109	0.414

#### Satisfaction distribution

**Fig. 3:**
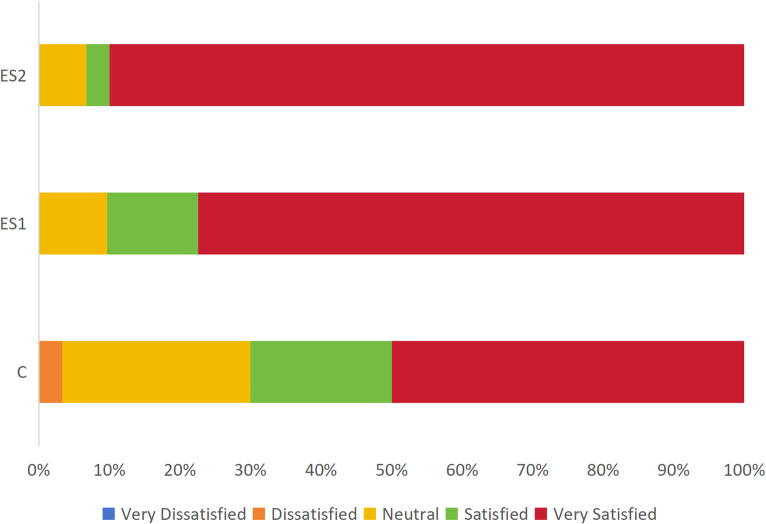
Patient satisfaction distribution across groups. χ^2^ = 12.669, *p* = 0.002.

As shown in Fig. 3, the distribution of satisfaction levels differed significantly among the three groups (χ^2^= 12.669, *p* = 0.002). The proportion of patients reporting “very satisfied” was markedly higher in the esketamine groups (ES1: 77.4%, ES2: 90.0%) compared to the control group (C: 50.0%). Conversely, the control group had a higher proportion of patients in the “dissatisfied” or “neutral” categories. Detailed numerical data are presented in Table 8.

**Table 8:** Patient satisfaction distribution.

Group	Very dissatisfied	Dissatisfied	Neutral	Satisfied	Very Satisfied
C	0 (0%)	1 (3.3%)	8 (26.7%)	6 (20.0%)	15 (50.0%)
ES1	0 (0%)	0 (0%)	3 (9.7%)	4 (12.9%)	24 (77.4%)
ES2	0 (0%)	0 (0%)	2 (6.7%)	1 (3.3%)	27 (90.0%)

χ^2^ = 12.669, *p* = 0.002.

### Correlation analysis between VAS and HAMD

To explore whether the observed improvement in mood was directly related to analgesic effects, we performed correlation analyses between 24-hour postoperative pain scores (VAS) and HAMD scores. As shown in Table 9, no significant correlations were observed in the total population between resting VAS and HAMD scores (Kendall’s tau-b = − 0.080, *p* = 0.336) or between activity VAS and HAMD scores (Kendall’s tau-b = − 0.024, *p* = 0.767).

Similarly, no significant correlations were observed in the within-group analyses.

Collectively, these findings indicate that the mood-improving effects of esketamine are largely independent of its analgesic properties, supporting a direct pharmacological action on emotional regulation.

**Table 9:** Correlation between 24-hour postoperative VAS and HAMD scores.

Population	VAS Rest vs. HAMD		VAS Activity vs. HAMD	
	tau-b	*p*-value	tau-b	*p*-value
Total Population	-0.080	0.336	-0.024	0.767
C	-0.090	0.543	0.079	0.592
ES1	0.027	0.855	-0.058	0.683
ES2	0.292	0.052	0.117	0.422

Kendall’s tau-b correlation coefficients are presented. No statistically significant correlations were observed (*p* < 0.05).

## Discussion

While our findings demonstrate significant improvement in mood outcomes, it is important to clarify the terminology used in this study. According to DSM-5 criteria, a formal diagnosis of ‘depression’ typically requires symptoms to persist for at least two weeks. The emotional changes observed within 24 to 72 hours postoperatively in our study are more accurately defined as ‘postoperative depressive state’ or ‘acute emotional distress’. Therefore, our results should be interpreted as esketamine’s effect on acute postoperative mood disturbances rather than clinically diagnosed depression. This distinction is crucial for accurate interpretation of our findings and aligns with the acute observation period of our study.

The findings of this randomized controlled trial demonstrate that the addition of esketamine to sufentanil-based PCIA significantly alleviates postoperative depression and anxiety state in elderly patients undergoing colorectal cancer resection, while maintaining comparable analgesic efficacy and safety to sufentanil alone. These results align with emerging evidence supporting the role of NMDA receptor antagonists in mood regulation and perioperative mental health optimization. Below, we contextualize these findings within existing literature and explore their clinical implications.

### Mechanistic insights: NMDA antagonism and mood enhancement

The antidepressant effects of esketamine observed in this study are likely mediated by its antagonistic action on NMDA receptors, which modulates glutamatergic neurotransmission and synaptic plasticity. Preclinical studies have shown that NMDA receptor blockade disinhibits GABAergic interneurons, leading to enhanced glutamate release in the prefrontal cortex and subsequent activation of neurotrophic signaling pathways, including BDNF synthesis^9,10,12,13^. These mechanisms are thought to facilitate rapid functional changes in limbic circuits that contribute to mood stabilization, although structural synaptic remodeling within this acute postoperative timeframe remains to be directly demonstrated in human subjects. Clinically, esketamine’s efficacy in reducing HAMD and HAMA scores within 24–72 h post-surgery mirrors findings from trials in non-surgical depression models, suggesting a shared neurobiological pathway^11,14,15^. Notably, the lack of dose-dependent effects between ES1 and ES2 groups implies that even low-dose esketamine (1 mg/kg) may suffice to initiate these neuroadaptive changes in elderly patients, potentially reducing risks associated with higher doses.

### Analgesic efficacy: divergence from prior studies

Contrary to previous reports where ketamine adjuncts enhanced postoperative analgesia, our study found no significant differences in VAS scores or rescue analgesia requirements between esketamine and control groups. This discrepancy may stem from the relatively low esketamine infusion rate (0.015–0.03 mg/kg/h), which falls below the recommended threshold (0.125–0.25 mg/kg/h) for synergistic opioid-sparing effects^16^. While subtherapeutic dosing likely limited analgesic benefits, it did not compromise esketamine’s antidepressant properties. The independence of esketamine’s mood-improving effects from its analgesic properties was further supported by correlation analyses, which revealed no significant associations between postoperative pain scores and HAMD scores in the overall population or within individual groups. These results align with the dissociation between analgesic and antidepressant outcomes observed in our primary analysis.This dissociation between analgesic and antidepressant outcomes may suggest the distinct mechanisms underlying pain and mood modulation, with the latter requiring lower NMDA receptor occupancy.

### Patient satisfaction: beyond pain relief

Despite comparable pain control, satisfaction rates were markedly higher in esketamine groups (ES1: 77.4%; ES2: 90% vs. C: 50%). This paradox may reflect esketamine’s ability to mitigate the psychological burden of surgery, such as existential distress or fear of recurrence, which are poorly addressed by opioids alone. Enhanced satisfaction could also arise from reduced opioid-related side effects (e.g., sedation), though our data showed no significant intergroup differences in adverse events. Future qualitative studies are warranted to explore patient perceptions of esketamine’s holistic benefits.

Esketamine significantly improved postoperative mood and patient satisfaction, this did not translate into shortened hospital stay. Several factors may explain this discrepancy. First, hospital discharge following colorectal cancer surgery is typically determined by multiple clinical parameters including bowel function recovery, surgical wound healing, return of oral intake, and resolution of surgical complications, which may be less sensitive to mood improvements within the acute postoperative period. Second, our study may have been underpowered to detect differences in hospital stay, as length of stay can be influenced by numerous confounding factors including institutional discharge protocols, social support availability, and patient preferences.

### Clinical implications and safety profile

The integration of esketamine into PCIA protocols offers a dual advantage: alleviating Postoperative Depression state without compromising respiratory stability—a critical consideration in elderly populations with heightened opioid sensitivity. The absence of dose-related safety concerns (e.g., hallucinations, hypertension) further supports its feasibility in geriatric care. However, clinicians should remain vigilant for rare neuropsychiatric reactions, particularly in patients with undiagnosed cognitive impairments.

### Limitations and future directions

This study has several limitations. First, the single-center design and modest sample size limit generalizability. Second, the follow-up period (72 h) precludes assessment of long-term mental health outcomes, which are essential given the chronicity of cancer-related depression. Third, the assessment of postoperative depression and anxiety was solely based on the HAMD and HAMA scales; while these scales are validated tools, they entail a certain degree of subjective dependence, fail to fully capture the multidimensional nature of postoperative psychological distress, and no objective physiological signal detection was incorporated in the present study. In recent years, electroencephalogram (EEG)-based depression recognition models (e.g., UA-DAAN, WDANet)^17,18^ and multimodal data fusion strategies (e.g., MF2-Net, EmoSavior)^19,20^ have exhibited remarkable advantages in depressive state identification. Future studies could integrate objective physiological signals such as electroencephalogram and heart rate variability with scale scores to construct a multidimensional assessment system, thereby improving the objectivity and accuracy of postoperative depressive state evaluation. Moreover, future multicenter trials with extended observation periods and mixed-method assessments (e.g., biomarkers, patient narratives) are needed to validate these findings. Fourth, the HAMD−17 assessment typically requires 20–30 min to complete. Conducting this evaluation at 24 h postoperatively may have introduced scoring bias due to patient fatigue, discomfort, or reduced cooperation. However, all assessments were performed by trained researchers who monitored patient comfort throughout and allowed for breaks when needed. Additionally, the consistency of findings across both the HAMD and HAMA scales, along with the similar pattern of results at 72 h postoperatively when patients were more recovered, suggests that any potential bias from patient cooperation was likely minimal.

## Data Availability

The original contributions presented in this study are included in the article, further inquiries can be directed to the corresponding authors.
